# Validation of deep learning natural language processing algorithm for keyword extraction from pathology reports in electronic health records

**DOI:** 10.1038/s41598-020-77258-w

**Published:** 2020-11-20

**Authors:** Yoojoong Kim, Jeong Hyeon Lee, Sunho Choi, Jeong Moon Lee, Jong-Ho Kim, Junhee Seok, Hyung Joon Joo

**Affiliations:** 1 School of Electrical Engineering, Korea University, Seoul, South Korea; 2 Department of Pathology, Korea University College of Medicine, Seoul, South Korea; 3 Korea University Research Institute for Medical Bigdata Science, Korea University, Seoul, Korea; 4 Department of Cardiology, Cardiovascular Center, Korea University College of Medicine, Seoul, South Korea

**Keywords:** Biomedical engineering, Data mining, Machine learning

## Abstract

Pathology reports contain the essential data for both clinical and research purposes. However, the extraction of meaningful, qualitative data from the original document is difficult due to the narrative and complex nature of such reports. Keyword extraction for pathology reports is necessary to summarize the informative text and reduce intensive time consumption. In this study, we employed a deep learning model for the natural language process to extract keywords from pathology reports and presented the supervised keyword extraction algorithm. We considered three types of pathological keywords, namely specimen, procedure, and pathology types. We compared the performance of the present algorithm with the conventional keyword extraction methods on the 3115 pathology reports that were manually labeled by professional pathologists. Additionally, we applied the present algorithm to 36,014 unlabeled pathology reports and analysed the extracted keywords with biomedical vocabulary sets. The results demonstrated the suitability of our model for practical application in extracting important data from pathology reports.

## Introduction

The pathology report is the fundamental evidence for the diagnosis of a patient. All kinds of specimens from all operations and biopsy procedures are examined and described in the pathology report by the pathologist. As a document that contains detailed pathological information, the pathology report is required in all clinical departments of the hospital. However, the extraction and generation of research data from the original document are extremely challenging mainly due to the narrative nature of the pathology report. As such, the data management of pathology reports tends to be excessively time consuming and requires tremendous effort and cost owing to its presentation as a narrative document.

Several conventional keyword extraction algorithms were carried out based on the feature of a text such as term frequency-inverse document frequency, word offset^1,2^. This approach is straightforward but not suitable for analysing the complex structure of a text and achieving high extraction performance.

Rule-based algorithms have been selectively adopted for automated data extraction from highly structured text data^3^. However, this kind of approach is difficult to apply to complex data such as those in the pathology report and hardly used in hospitals. The advances in machine learning (ML) algorithms bring a new vision for more accurate and concise processing of complex data. ML algorithms can be applied to text, images, audio, and any other types of data.

The most widely used ML approach is the support-vector machine, followed by naïve Bayes, conditional random fields, and random forests^4^. Deep learning approaches are increasingly adopted in medical research. For example, long short-term memory (LSTM) and convolutional neural networks (CNN) were carried out for named entity recognition in biomedical context^5,6^.

There have been many studies for word embeddings to deal with natural language in terms of numeric computation. In conventional word embedding, a word can be represented by the numeric vector designed to consider relative word meaning as known as word2vec^7^. In other aspects, the word tokenizing technique is used to handle rarely observed words in the corpus^8^. Also, the pre-trained word representation is widely conducted for deep learning model such as contextual embedding^9^, positional embedding, and segment embedding^10^.

The bidirectional encoder representations from transformers (BERT) model is one of the latest deep learning language models based on attention mechanisms^10^. It has been applied in many kinds of biomedical natural language processing (NLP) research, including clinical entity normalization, text mining (i.e., BioBERT), breast cancer concept extraction, and discharge summary diagnosis extraction^11–14^.

The present study aimed to develop a keyword (specimen, procedure, pathologic diagnosis) extraction model for free-text pathology reports from all clinical departments.

## Results

### Experimental setup

We employed a pre-trained BERT that consisted of 12 layers, 768 hidden sizes, 12 self-attention heads, and an output layer with four nodes for extracting keywords from pathology reports. BERT followed two types of pre-training methods that consist of the masked language model and the next sentence prediction problems^10^. In the masked language model, 15% of the masked word was applied on an optimized strategy. In the next sentence prediction, two sentences are given, and then the model learns to classify whether the sentences are precedent relation. The BooksCorpus dataset^15^ and English Wikipedia were used to apply these pre-training methods. In our experiment, we used Adam with a learning rate of 2e^-5^ and a batch size of 16.

A total of 6771 pathology reports were analysed for keyword extraction. Each report included the three types of keywords that professional pathologists manually extracted: specimen, procedure, and pathology. Specimen indicated the organ and region to test; procedure, the procedure used to test; and pathology, the pathological result. The keywords were included in the original text but did not necessarily appear in adjacent phrases.

Of the 6771 pathology reports, 6093 were used to train the model, and 678 were used to evaluate the model for pathological keyword extraction. The training set and test set were randomly split from 6771 pathology reports after paragraph separation. Each dataset included the original text that represented the results of the pathological tests and corresponding keywords. Table 1 shows the number of unique keywords for each type in the training and test sets. Compared with conventional keyword extraction, both datasets had fewer unique keywords, which we presumed to be due to the redundancy in keywords for patients who had similar symptoms, leading to an over-estimated performance.

**Table 1:** Number of unique keywords in the pathology report dataset.

Dataset	Type	#
Training	Specimen	619
	Procedure	227
	Pathology	733
Test	Specimen	200
	Procedure	106
	Pathology	202

### Fine-tuning

**Figure 1:**
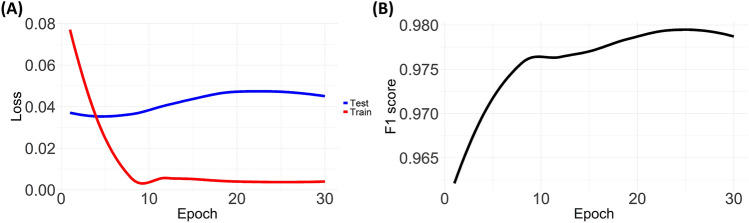
Fine-tuning for the keyword extraction of pathology reports (**A**) Cross-entropy loss on the training and test sets according to the training step (**B**) F1 score on the test set according to the training step.

We investigated the optimization process of the model in the training procedure, which is shown in Fig. 1. Figure 1A shows the training/test loss. Training loss was calculated by accumulating the cross-entropy in the training process for a single mini-batch. Meanwhile, test loss was calculated after completing the training. Both losses were rapidly reduced until the 10th epoch, after which the loss increased slightly. Figure 1B presents the F1 score for keyword extraction. The F1 score was evaluated on the test set through training epochs. The F1 score rapidly increased until the 10th epoch. It continuously increased after the 10th epoch in contrast to the test loss, which showed a change of tendency. Thus, the performance of keyword extraction did not depend solely on the optimization of classification loss.

**Figure 2:**
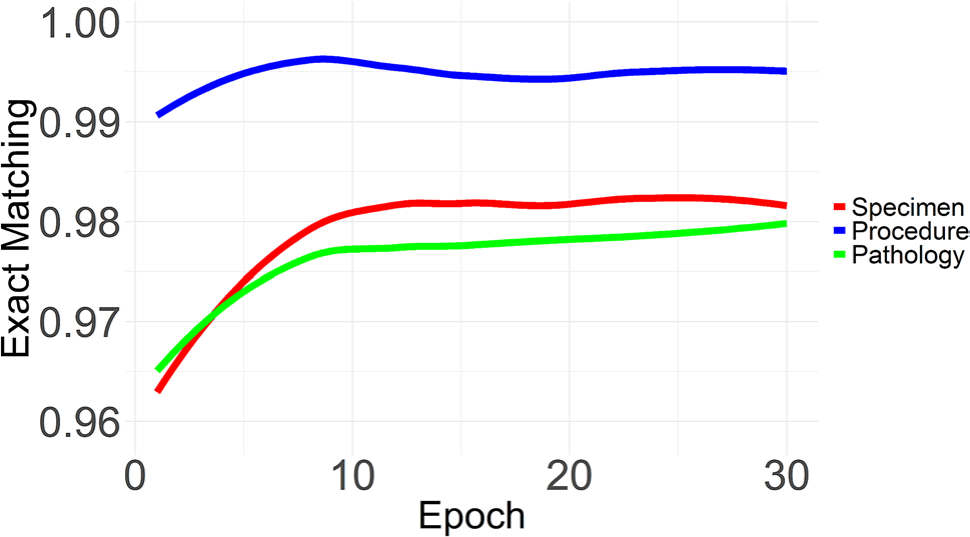
Exact matching for the three types of pathological keywords according to the training step.

Additionally, we evaluated the performance of keyword extraction for the three types of pathological domains according to the training epochs. Figure 2 depicts the exact matching rates of the keyword extraction using entire samples for each pathological type. The exact matching was measured on the test set. The extraction procedure showed an exact matching of 99% from the first epoch. The specimen and pathology were extracted over 96% from the first epoch. The overall extractions were stabilized from the 10th epoch and slightly changed after the 10th epoch.

**Figure 3:**
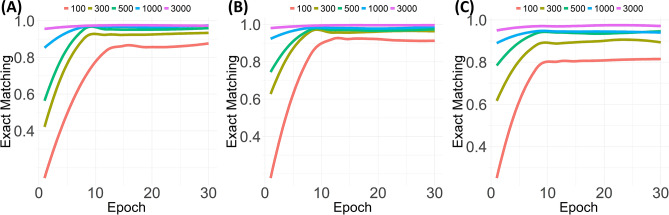
Exact matching rate for the three types of pathological keywords according to the number of samples used to train the Bidirectional Encoder Representations from Transformers model (**A**) Specimen type (**B**) Procedure type (**C**) Pathology type.

We also investigated the exact matching using different sample numbers to train the model, as shown in Fig. 3. We used 100, 300, 500, 1000, and 3000 samples to compare the dependency for the number of samples on the training of keyword extraction. The performance for the pathology type, among the keyword types, showed the most intensive dependency for sample numbers.

### Keyword extraction results

We compared the performance of five supervised keyword extraction methods for the pathology reports. The methods were two conventional deep learning approaches, the Bayes classifier, and the two feature-based keyphrase extractors named as Kea^2^ and Wingnus^1^. Performance was evaluated in terms of recall, precision, and exact matching. The deep learning methods (BERT, LSTM, CNN) were evaluated after the training of 30 epochs. All methods used the identical dataset that is pathology report as inputs.

We briefly introduce each keyword extraction model. One of the deep learning approaches was an LSTM-based model that consisted of an embedding layer, an LSTM layer, and a fully connected layer. Another was the CNN structure that consisted of an embedding layer, two convolutional layers with max pooling and drop-out, and two fully connected layers. A simple Bayes classifier was also used. These three models classified each word for the three keyword types. We also used Kea and Wingnus, which are feature-based candidate selection methods. These methods select keyphrase candidates based on the features of phrases and then calculate the score of the candidates. These were not suitable to distinguish keyword types, and as such, the three individual models were separately trained for keyword types.

Additionally, we carried out the pre-training of the LSTM model and the CNN model through the next sentence prediction^10^, respectively. The English Wikipedia dataset was used for pre-training. Text was only extracted from the dataset by ignoring lists, tables, headers. We organized pairs of two sentences that have precedent relation and then labeled these pairs as *IsNext*. For each pair, one sentence was randomly selected and matched with the next sentence. On the other hand, we randomly selected two sentences and labeled them as *NotNext*. In the pre-training, the ratio of the label was 33.3% of *IsNext* and 66.6% of *NotNext.* The pre-training was carried out for 150,000 sentence pairs until reaching at least 99% of accuracy.

Table 2 shows the keyword extraction performance of the seven competitive methods and BERT. Compared with the other methods, BERT achieved the highest precision, recall, and exact matching on all keyword types. It showed a remarkable performance of over 99% precision and recall for all keyword types. It also correctly extracted the procedure type by 99.56%. Similarly, the performance of the two conventional deep learning models with and without pre-training was outstanding and only slightly lower than that of BERT. The pre-trained LSTM and CNN models showed higher performance than the models without pre-training. The pre-trained models achieved sufficient high precision and recall even compared with BERT. However, the models showed lower exact matching than BERT. The Bayes classifier showed poor performance only for exact matching because it is not suitable for considering the dependency on the position of a word for keyword classification. Meanwhile, the two keyphrase extractors showed the lowest performance. These extractors did not create proper keyphrase candidates and only provided a single keyphrase that had the maximum score. The difference in medical terms and common expressions also reduced the performance of the extractors.

**Table 2:** Summary of keyword extraction performance for pathology reports.

Methods	Precision			Recall			Exact Matching		
	SPE	PRO	PAT	SPE	PRO	PAT	SPE	PRO	PAT
BERT	0.9951	0.9985	0.9961	0.9962	0.9990	0.9938	0.9839	0.9956	0.9795
LSTM	0.9871	0.9932	0.9438	0.9764	0.9919	0.9387	0.9327	0.9868	0.9151
Pre-trained LSTM	0.9940	0.9978	0.9924	0.9915	0.9979	0.9934	0.9646	0.9794	0.9631
CNN	0.9740	0.9769	0.9320	0.9716	0.9758	0.9204	0.9327	0.9502	0.8770
Pre-trained CNN	0.9947	0.9958	0.9855	0.9903	0.9964	0.9823	0.9631	0.9690	0.9218
Bayes Classifier	0.9300	0.9601	0.8956	0.8946	0.9775	0.8227	0.7130	0.9078	0.5168
Kea	0.7321	0.1154	0.3499	0.3751	0.1076	0.1198	0.1010	0.0981	0.0190
WINGNUS	0.6227	0.1786	0.1552	0.3904	0.1650	0.1017	0.1098	0.1552	0.0835

SPE represents specimen type, PRO represents procedure type, and PAT represents pathology type.

This experiment was carried out in python on 24 CPU cores, which are Intel (R) Xeon (R) E5-2630v2 @ 2.60 GHz, 128 GB RAM, and GTX 1080Ti. The times elapsed for training each model are summarized in Table 3. Especially, we listed the average running time for each epoch of BERT, LSTM, and CNN.

**Table 3:** Running times for model training.

Methods	Times (s)
BERT (1 epoch)	19.0
LSTM (1 epoch)	127.8
CNN (1 epoch)	32.0
Bayes Classifier	2.4
Kea	13,081.1
WINGNUS	10,815.5

### Similarity in standard medical vocabulary

To investigate the potential applicability of the keyword extraction by BERT, we analysed the similarity between the extracted keywords and standard medical vocabulary. We extracted 65,024 specimen, 65,251 procedure, and 65,215 pathology keywords by BERT from 36,014 reports that were not used to train or test the model. After removing the duplicates, we prepared unique keyword sets. As the standard vocabulary adopted the combined terms of specimen and pathologic diagnoses (e.g., adenocarcinoma of sigmoid colon), we also built a combined vocabulary of specimen and pathologic diagnoses from the extracted keywords as shown in Table 4.

**Table 4:** Unique set of extracted keywords from unlabeled pathology reports.

Type	#
Specimen	3052
Pathology	3475
Specimen + Pathology	9084
Procedure	797

Examples of standard medical vocabulary include the International Classification of Disease, Systemized nomenclature of clinical medicine terms, North American Association of Central Cancer Registries (NAACCR), and medical subject headings (MeSH)^16,17^. Meanwhile, there is no well-known vocabulary specific to the pathology area. As such, we selected NAACCR and MeSH to cover both cancer-specific and generalized medical terms in the present study. Almost all clinical cancer registries in the United States and Canada have adopted the NAACCR standard^18^. A recently developed biomedical word embedding set, called BioWordVec, adopts MeSH terms^19^. In filtering invalid and non-standard vocabulary, 24,142 NAACCR and 13,114 MeSH terms were refined for proper validation.

The extracted pathology keywords were compared with each medical vocabulary set via Wu–Palmer word similarity, which measures the least distance between two word senses in the taxonomy with identical part-of-speech^20^. We measured the similarity between the extracted keyword and the medical vocabulary by averaging the non-zero Wu–Palmer similarity and then selecting the maximum of the average.

**Figure 4:**
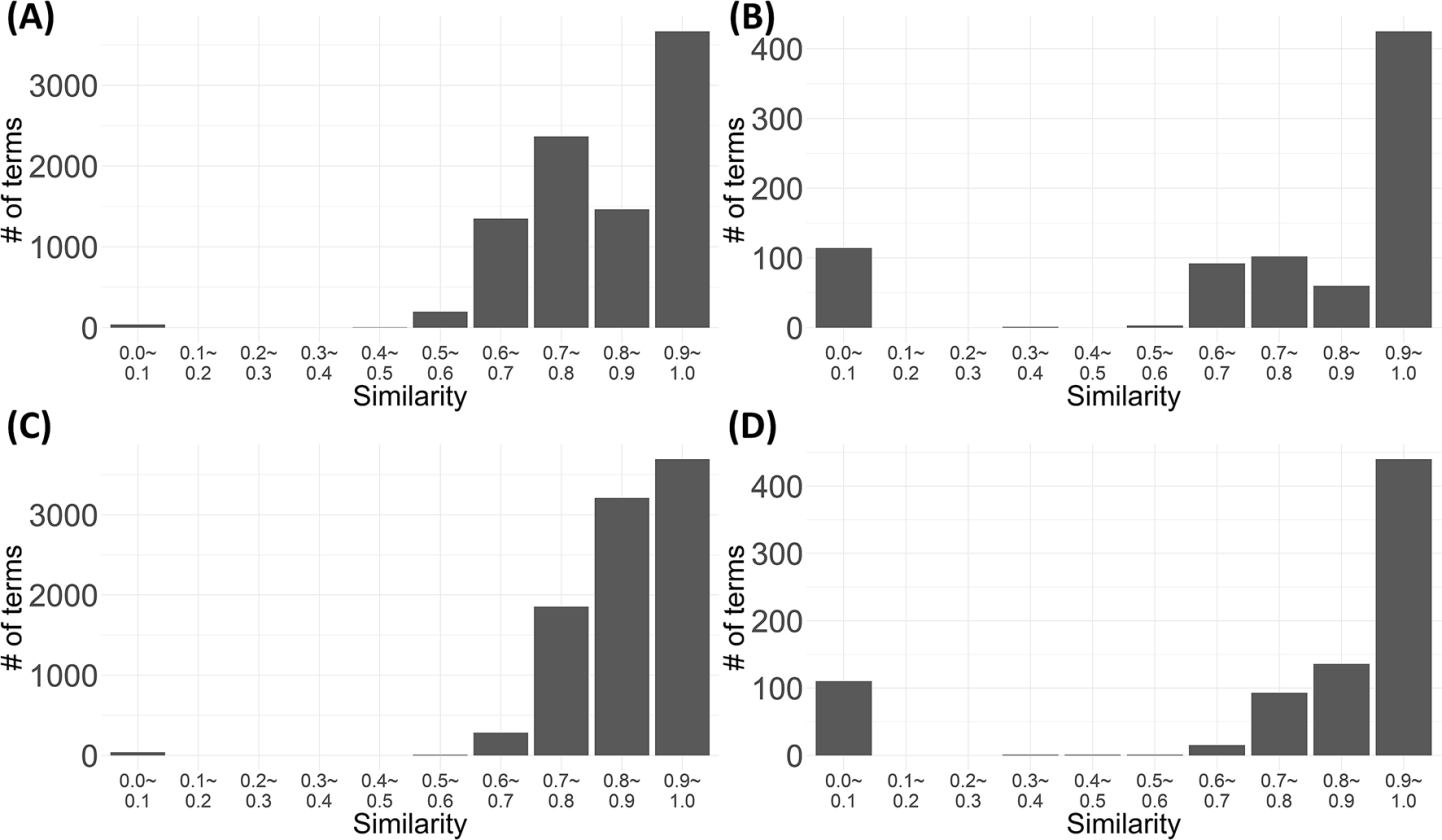
Distribution for the maximum value of word similarity for each extracted keyword and the existing pathology vocabulary (**A**) Specimen + Pathology type and medical subject headings (MeSH) (**B**) Procedure type and MeSH (**C**) Specimen + Pathology type and North American Association of Central Cancer Registries (NAACCR) (**D**) Procedure type and NAACCR.

Figure 4 shows the distribution of the similarity between the extracted keywords and each medical vocabulary set. The majority of the specimen + pathology type terms related strongly to two vocabulary sets. Similarly, the procedure type showed a distribution skewed to the right. For the procedure type, 114 and 110 zero similarities were estimated for MeSH and NAACCR among the 797 extracted keywords, respectively. For the specimen + pathology type, we found 38 zero similarities compared with both vocabulary sets among 9084 extracted keywords. The keywords that showed zero similarity included terms that were incorrectly extracted, terms with no relation with such vocabulary sets, and terms extracted from typos. Our model managed to extract the proper keywords from the misrepresented text.

## Discussion

In this work, we proposed a keyword extraction method for pathology reports based on the deep learning approach. We employed one of the recent deep learning models for NLP, BERT, to extract pathological keywords, namely specimen, procedure, and pathology, from pathology reports. We evaluated the performance of the proposed algorithm and five competitive keyword extraction methods using a real dataset that consisted of pairs of narrative pathology reports and their pathological keywords. In addition to the evaluation, we applied the present algorithm to unlabeled pathology reports to extract keywords and then investigated the word similarity of the extracted keywords with existing biomedical vocabulary. The results supported the usefulness of the present algorithm. An advantage of the present algorithm is that it can be applied to all pathology reports of benign lesions (including normal tissue) as well as of cancers.

Many NLP systems for extracting clinical information have been developed, such as a lymphoma classification tool^21^, a cancer notifications extracting system^22^, and a biomarker profile extraction tool^23^. These authors adopted a rule-based approach and focused on a few clinical specialties. However, the inter-institutional heterogeneity of the pathology report format and vocabulary could restrict generalizability in applying pipelines.

Rapid progress in ML technologies has accelerated the progress in this field and specifically allowed our method to encompass previous milestones. Yala et al. adopted Boostexter (a boosting classification system) to parse breast pathology reports^24^. Our work adopted a deep learning approach more advanced than a rule-based mechanism and dealt with a larger variety of pathologic terms compared with restricted extraction. Leyh-Bannurah et al. developed a key oncologic information extraction tool confined for prostate cancer^25^. Our method is suitable for dealing with overall organs, as opposed to merely the target organ. Oliwa et al. developed an ML-based model using named-entity recognition to extract specimen attributes^26^. Our model could extract not only specimen keywords but procedure and pathology ones as well. Giannaris et al. recently developed an artificial intelligence-driven structurization tool for pathology reports^27^. Our work aimed at extracting pathological keywords; it could retrieve more condensed attributes than general named entity recognition on reports.

Many deep learning models have been adopted for keyword extraction for free text. Cheng and Lapata proposed a data-driven neural summarization mechanism with sentence extraction and word extraction using recurrent and convolutional network structure^28^. However, our model showed outstanding performance compared with the competitive LSTM model that is similar to the structure used for the word extraction. Zhang et al. suggested a joint-layer recurrent neural network structure for finding keyword^29^. They employed a dual network before the output layer, but the network is significantly shallow to deal with language representation. Zhang et al. developed a target-centered LSTM model^30^. This model classifies whether a single word is a keyword. It is prone to errors of extracting not exactly matched keyword rather than our model that extracts keywords in one step. These deep learning models used a unidirectional structure and a single process to train. In contrast, our model adopted bidirectional representations and pre-training/fine-tuning approaches.

Several NLP studies on electronic health records have attempted to create models that accomplish multiple tasks based on an advanced deep learning approach. Li et al. developed a BERT-based model for electronic health records (EhrBERT) to normalize biomedical entities on the standard vocabulary^11^. In comparison, our model could extract pathological keywords stated in the original text and intuitively summarize narrative reports while preserving the intention of the vocabulary. Lee et al. proposed an adjusted BERT that is additionally pre-trained with biomedical materials (bioBERT)^12^ and employed it to perform representative NLP tasks. Meanwhile, our algorithm could not only extract but also classify word-level keywords for three categories of the pathological domain in the narrative text. Chen et al. proposed a modified BERT for character-level summarization to reduce substantial computational complexity^14^. In contrast, our algorithm could perform word-level keyword extraction because of restrictive vocabulary usage in the pathological domain, thereby requiring a shorter sequence for the same text and reducing computational load.

The present study has several limitations. First, the algorithm was developed using the pathology reports of a single institution, which might limit the generalizability of its application to other institutions. However, we are constantly upgrading our algorithm. Recently, it was further trained with different types of pathology reports that were generated manually. Second, other information, including pathologic staging and tumor size, were not considered in the present algorithm. The extraction of these data through the algorithm would be helpful in building pathology big data that contain abundant information in the future. Third, clinical guidelines and practice constantly change. When new guidelines with new procedures and diagnoses are implemented, the performance of the algorithm could be affected, and thus, the algorithm should be recalibrated. Fourth, the present algorithm did not show 100% extraction accuracy. However, it demonstrated high (> 90%) accuracy; the extent of this discrepancy could be compensated by statistical methods.

## Conclusion

The proposed keyword extraction model for pathology reports based on BERT was validated through performance comparison using electronic health records and practical keyword extraction of unlabeled reports. The present algorithm showed a significant performance gap with five competitive methods and adequate application results that contain proper keyword extraction from misrepresented reports. We expect that this work can be utilized by biomedical researchers or medical institutions to solve related problems.

## Materials and methods

### Data approval

This study was created with the hospital common data model database construction process. The study protocol was approved by the institutional review board of Korea University Anam Hospital (IRB NO. 2019AN0227). Written informed consent was waived by the institutional review board of Korea University Anam Hospital because of the use of a retrospective study design with minimal risk to participants. The study also complied with the Declaration of Helsinki.

### Pathology report data and preprocessing

The pathology reports were stored as a table in an electronic health records database. One cell in the ‘results’ column of the pathology dataset contained one pathology report. The name and identification code of the patients and pathologists were stored in separate columns. No names and identification codes were indicated in the ‘results’ column. We acquired the consecutive 39,129 pathology reports from 1 January to 31 December 2018. Among them, 3115 pathology reports were used to build the annotated data to develop the keyword extraction algorithm for pathology reports. The other 36,014 pathology reports were used to analyse the similarity of the extracted keywords with standard medical vocabulary, namely NAACCR and MeSH.

When one pathology report described more than two types of specimens, it was divided into separate reports according to the number of specimens. The separated reports were organized with double or more line breaks for pathologists to understand the structure of multiple texts included in a single pathology report. Several reports had an additional description for extra information, which was not considered to have keywords. The description was also organized with double or more line breaks and placed at the bottom of the report.

The pathology reports were divided into paragraphs to perform strict keyword extraction and then refined using a typical preprocess in NLP. Each pathology report was split into paragraphs for each specimen because reports often contained multiple specimens. After the division, all upper cases were converted to lowercase, and special characters were removed. However, numbers in the report were not removed for consistency with the keywords of the report. Then, each word was tokenized using WordPiece embeddings^8^. Finally, 6771 statements from 3115 pathology reports were used to develop the algorithm.

### Keyword extraction strategy and labeling

The present study focused on three types of keyword extraction tasks: (1) body organ, (2) procedure for specimen acquisition, and (3) final pathologic diagnosis. All keywords were extracted manually by a certified clinical data specialist and confirmed by a pathologist.

Each word piece in the reports was assigned one of the keyword classes through the labeled keywords. The body organ of a specimen was mapped as **specimen**. The procedure used to acquire the sample was mapped as **procedure**. The pathological decision was mapped as **pathology**. Lastly, all of the remaining words were assigned **O,** representing ‘otherwise.’ Accordingly, tokens split by the tokenizer were linked with the tag of words, as well.

**Figure 5:**
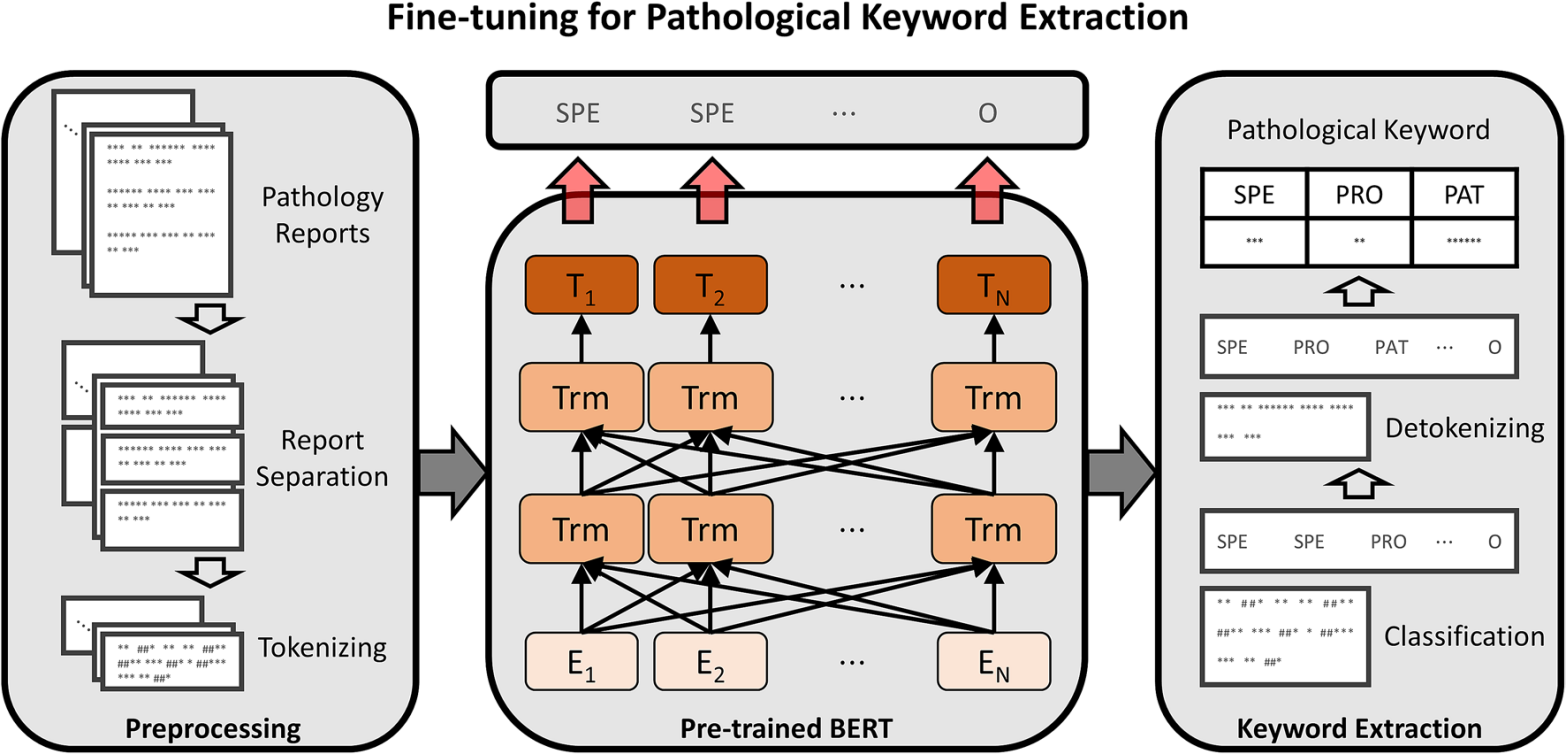
Keyword extraction algorithm based on Bidirectional Encoder Representations from Transformers for pathology reports. SPE represents specimen type, PRO represents procedure type, and PAT represents pathology type.

In this work, a pre-trained BERT^10^ was employed and fine-tuned for pathology reports with the keywords, as shown in Fig. 5. The model classified the token of reports according to the pathological keyword classes or otherwise. To tag the keyword classes of tokens, we added the classification layer of four nodes to the last layer of the model. Accordingly, the cross-entropy loss was used for training the model.

After the classification, the tagged tokens were detokenized through estimated probability via the model. Several sets of tokens were combined into one word. Let ${w}_{i}$ be the $i$th word in a pathology report and ${t}_{ij}$ be $j$th the token of ${w}_{i}$. The probability of pathological keyword classification for ${w}_{i}$ was estimated by.

$${w}_{i}^{l}= \frac{{\sum }_{j=1}^{J}\mathrm{exp}\left({p}_{ij}^{l}\right)}{{\sum }_{j=1}^{J}\sum_{l=1}^{4}\mathrm{exp}\left({p}_{ij}^{l}\right)}$$

where $l$ denotes pathological class and ${p}_{ij}^{l}$ denotes the output of the model before the softmax function for token ${t}_{ij}$. Lastly, the pathological class of ${w}_{i}$ was determined by $\underset{l}{\mathrm{argmax}}{w}_{i}^{l}$.

## Data Availability

Source code is available at https://github.com/KU-RIAS/Keyword-Extraction-for-Pathology-Reports-with-BERT.
